# Intrapulmonary mature cystic teratoma of the lung: case report of a rare entity

**DOI:** 10.1186/s12893-020-00864-y

**Published:** 2020-09-14

**Authors:** Parviz Mardani, Reyhaneh Naseri, Armin Amirian, Reza Shahriarirad, Mohammad Hossein Anbardar, Damoun Fouladi, Keivan Ranjbar

**Affiliations:** 1 Thoracic and Vascular Surgery Research Center, Shiraz University of Medical Science, Shiraz, Iran; 2 Department of Surgery, Shiraz University of Medical Sciences, Shiraz, Iran; 3 Student Research Committee, Shiraz University of Medical Sciences, Shiraz, Iran; 4 Department of Pathology, Namazee Teaching Hospital, School of Medicine, Shiraz University of Medical Sciences, Shiraz, Iran

**Keywords:** Teratoma, Lung, Case report, Pathology, Surgery

## Abstract

**Background:**

Intrapulmonary teratoma (IPT) is a rare type of extra gonadal teratoma which often presents with non-specific symptoms and can be misdiagnosed as other diseases. Here we report a patient with IPT which was initially misdiagnosed as lung hydatid cyst versus abscess.

**Case presentation:**

We report an intrapulmonary teratoma in a 27-year-old female presenting with persistent chest pain and dyspnea since a few years prior to her admission with associated symptoms of cough and fever. Chest x-ray only showed left side massive pleural effusion and computed tomography scan of the lungs was suggestive of hydatid cyst or a lung abscess. She underwent lobectomy and postoperative histopathological study revealed IPT as the final diagnosis.

**Conclusion:**

Due to the non-specific symptoms and rarity, IPT can be easily misdiagnosed at first. It is essential that physicians take into account the possibility of IPT when approaching a new case of lung mass.

## Background

Teratomas are benign germ cell tumors that are mostly found in the gonads with a low malignant transformation potency [1–3]. Extra-gonadal germ cell tumors are considered rare with mediastinum as the most common site [4], but can also arise in other areas such as the head and neck [5], retroperitoneum, sacrococcygeal region and on rare occasions the lung, which is considered as an intrapulmonary teratoma (IPT) [6]. In this study, we present a rare case of a benign intrapulmonary teratoma in a 27-year-old female involving the left upper lobe of the lung, which was successfully treated by lobectomy with no recurrence during a 6 year follow-up.

## Case presentation

A 27-year-old female visited our clinic with an unremarkable past medical history, with the chief complaint of progressive dyspnea and chest pain since 2 weeks prior to admission, which was recently accompanied by non-productive cough, chills, fever, and orthopnea. She also reported a mild, episodic, and occasionally pleuritic chest pain that radiated to back and left upper extremity. She denied any nausea, vomiting, rashes, joint pain, weight loss, or a history of smoking. She also reported a previous admission a few years ago due to dyspnea and chest pain in which after normal cardiac evaluation, was discharged with no established diagnosis.

On physical examination, the patient was febrile (38.3 °C orally) and breathing sound was decreased in lower two-third of the left lung. Other systemic exams were unremarkable. The patient underwent radiological chest evaluation, in which chest X-ray revealed massive left side pleural effusion with no apparent focal opacities. On admission, routine blood investigations including renal and liver function tests were within normal limits, apart from white blood cell count that showed leukocytosis (14.8 × 10^3^). On the suspicion of rheumatologic disorders, rheumatoid factors were evaluated which were all normal. Subsequently, a chest computed tomography (CT) scan also revealed pleural effusion, which along with previous findings, provided us with the impression of empyema. Therefore, a pleural drainage needle catheter was inserted but due to insufficient drainage, it was replaced with a chest tube.

Considering patients persistent fever and CT scan revelations, a provisional diagnosis of hydatid cyst versus lung abscess was made, and she was administered different antibiotics (ceftriaxone 1 g intravenous every 12 h/clindamycin 600 mg intravenous every 8 h / Imipenem 500 mg intravenous every 6 h) and Albendazole (400 mg orally, daily), however, the symptoms did not alleviate and due to the possibility of hydatid cyst, intensive procedures such as aspiration and biopsy was avoided and surgical interventions to remove the lesion was suggested for treatment. Furthermore, abdominopelvic ultrasonography was done to rule out possible liver, retroperitoneal, and gonadal mass, which no significant findings were detected.

The patient was operated under general anesthesia. Due to the severe adhesions caused by the recurrent previous infections, the operation changed from up position thoracoscopy to posterolateral thoracotomy. While exploring the pleural cavity, a grayish multi-lobulated firm intrapulmonary cystic mass (13 × 11 × 4 cm) was detected in the left upper lobe which was filled with hair and keratinized material. Based on the severe involvement and the retraction of the left upper lobe, left upper lobectomy was carried out.

**Fig. 1:**
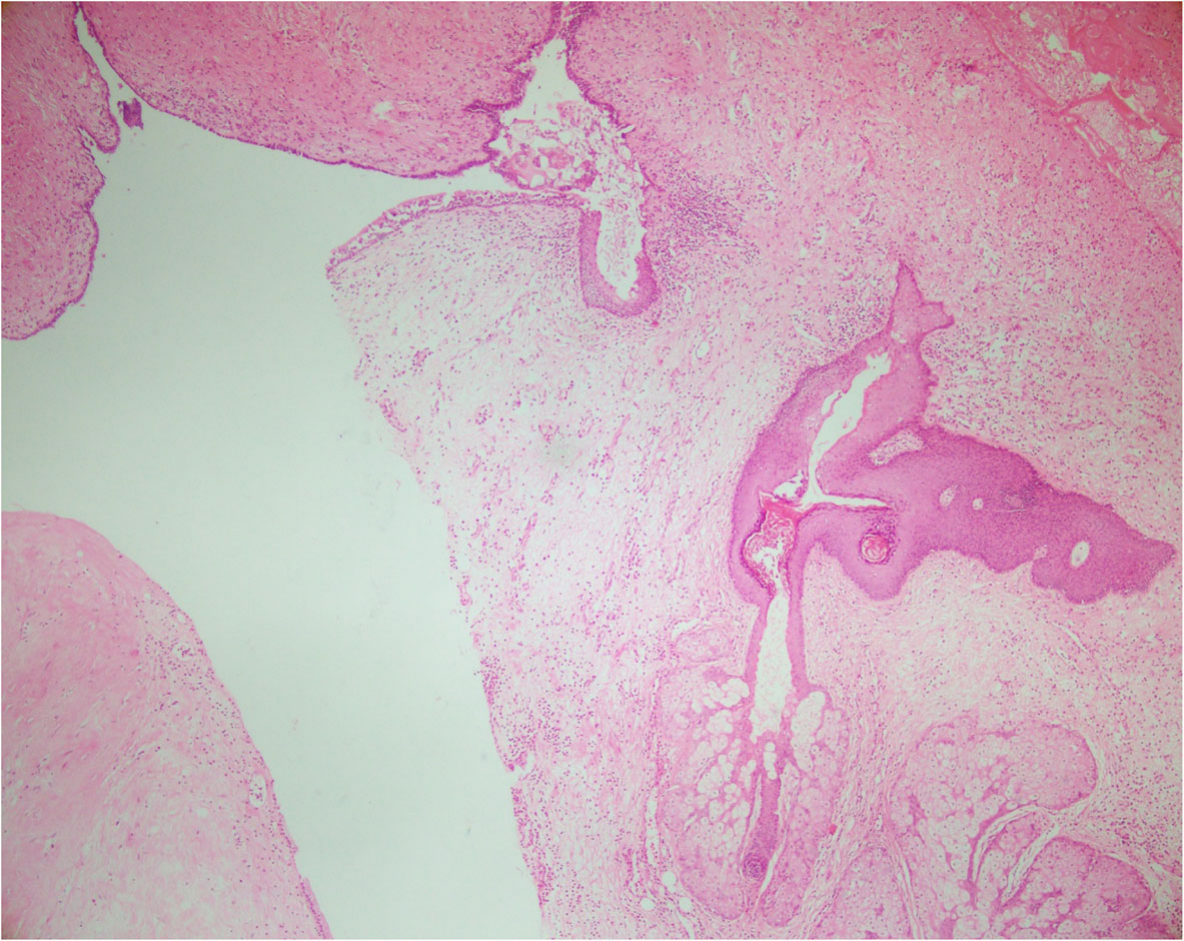
Microscopic section shows low power view of mature cystic teratoma consist of skin tissue, sebaceous gland and respiratory epithelium. (Hematoxyline and Eosin, × 40)

**Fig. 2:**
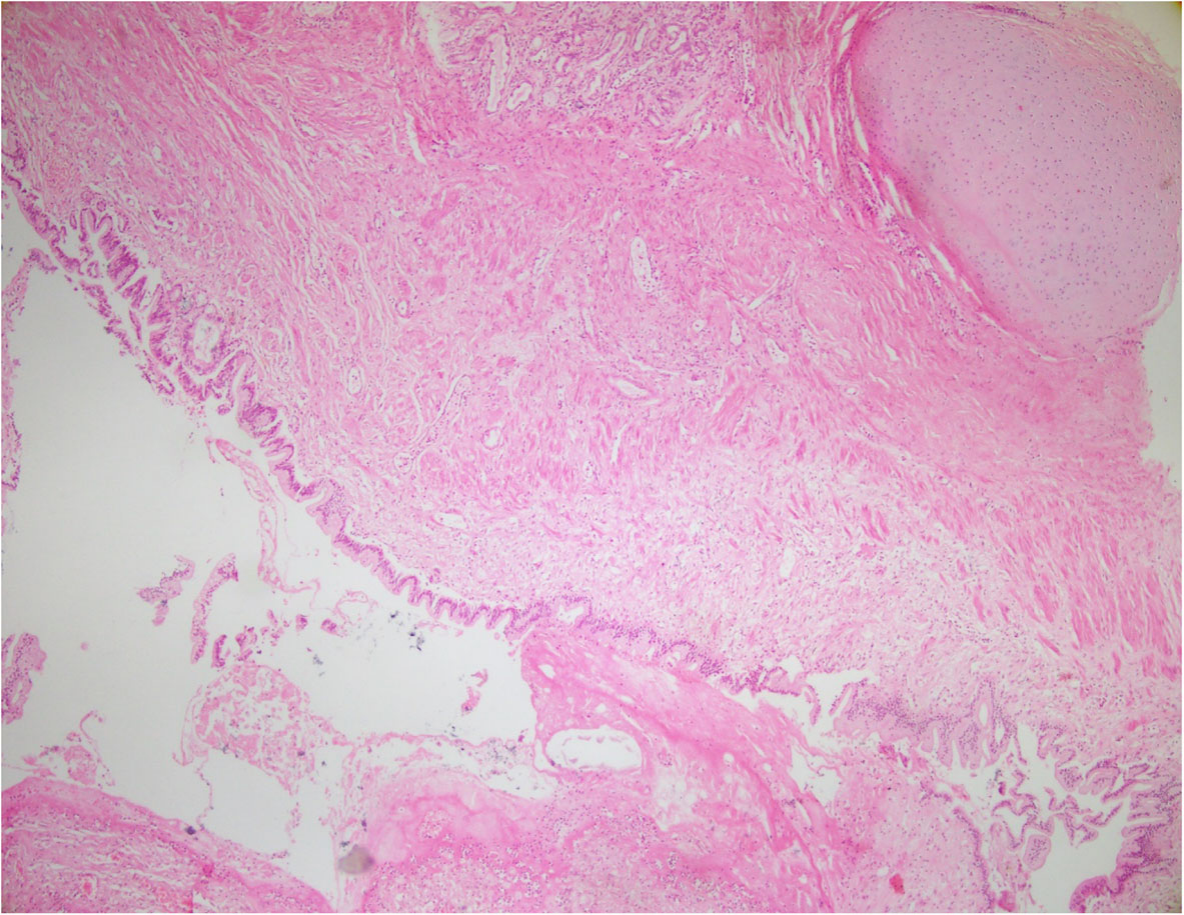
Microscopic section shows low power view of mature cystic teratoma consist of mature cartilage and mucinous epithelium. (Hematoxyline and Eosin, × 40)

**Fig. 3:**
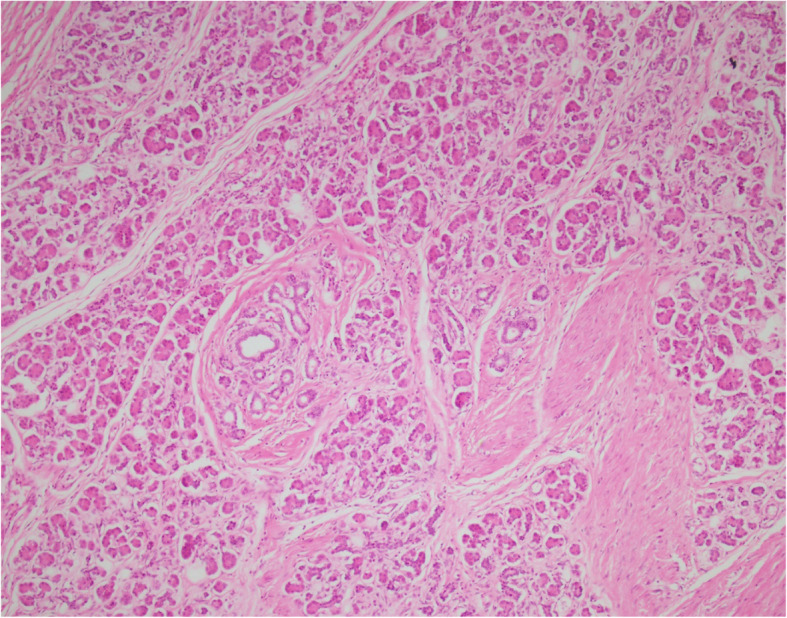
Microscopic section shows pancreatic tissue. (Hematoxyline and Eosin, × 100)

The specimen was collected from the lesion and the pleura for microscopic pathological evaluation. The gross examination of the operated specimen showed the upper lobe of the lung attached to a lobulated grayish cystic mass measuring 13 × 11 × 4 cm. Cut sections of the mass revealed unilocular cystic lesion filled with hair and waxy material. Microscopic sections showed the cystic lesion consisted of endodermal, ectodermal, and mesodermal components. Pancreatic tissue, mucinous epithelium, respiratory epithelium, epidermal tissue with sebaceous glands, adipose tissue, smooth muscle, and cartilage were identified in multiple microscopic sections (Figs. 1, 2, 3). Non-tumoral tissue showed pneumonia and pleural excision showed fibrinoid degeneration. No immature or malignant component was identified and the diagnosis of mature cystic teratoma was confirmed.

**Fig. 4:**
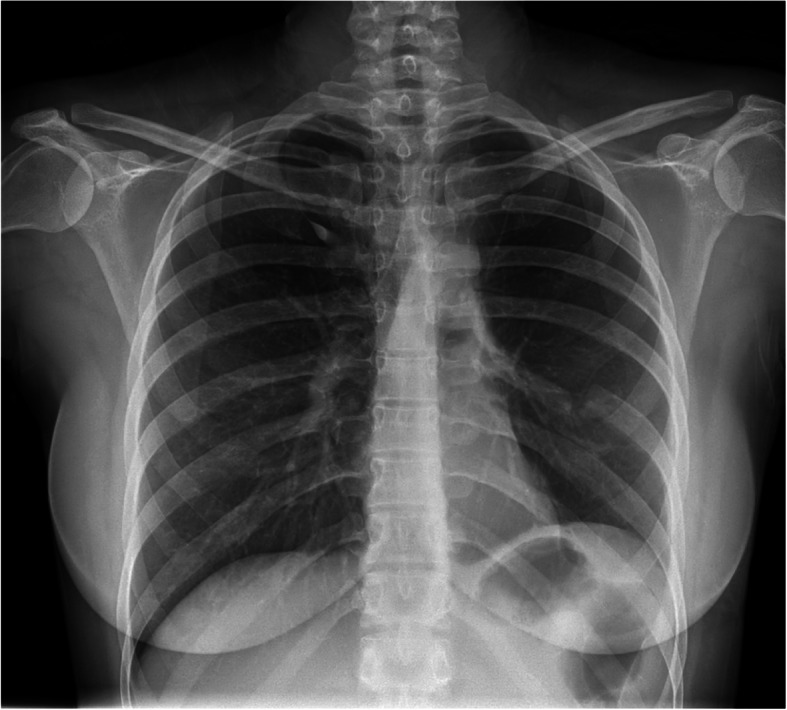
Follow-up chest x-ray of a 27-year-old female 4 month after left upper lobe lobectomy

Based on the patients’ surgical and histopathological findings, a final diagnosis of intrapulmonary mature cystic teratoma was achieved and the patient was discharged after 8 days with an uneventful post-op course. Follow up during the next years showed no sign of recurrence and a normal chest x-ray (Fig. 4).

## Discussion and conclusion

In this study, we presented a rare clinical case of IPT in a 27-year-old female involving the left upper lobe of the lung which presented with chest pain, dyspnea, low-grade fever, and dry cough. Teratomas are germinal cell tumors that are mostly present in gonads but can also be seen in extragonadal tissues [7]. IPT as a type of extragonadal teratoma is considered extremely rare, which can occur at any age but commonly due to their slow growth and voluminosity of the lung, presents mostly at the 3rd decade of life, similar to our presented case [8]. Regarding the tumor size, a review by Iwasaki et al. reported that the size varies in different cases, ranging from few centimeters up to 30 cm on the largest diameter [9].

Teratomas are usually considered benign tumors but in ovaries in 2% of the cases, they can undergo a malignant transformation [3]. until 2012, only 8 cases of malignant IPT have been reported. All the cases were male and the prognosis was generally poor with patients having a few days to months to live after presenting with symptoms. The malignant trasnformation can happen in any type of tissue present in the teratoma and is potensialy capable of metastasis to lymphnodes and other regions [1].

Previous studies have proved that there is an equal distribution of IPT by sex for men and women [8]. Due to indeterminate reasons, the predominant location of IPT is the upper lobe as similar to our case [10]. Patients become symptomatic due to the compression of the surrounding structures.

In our case, the patient presented with chest pain, dyspnea, fever, and dry cough. Similar studies have shown the common presenting clinical symptoms of IPT include chest pain as the most common presentation, fever, cough, dyspnea, and also hallmarks of pneumonia or bronchiectasis [8]. Trichoptysis (expectoration of hair) is a rare pathognomonic symptom that provides strong evidence in favor of IPT and usually occurs in the late course of the disease followed by tumor invasion into the tracheobronchial tree [11].

Reports from other studies have shown that laboratory tests are usually within normal limits [12, 13], which is aligned with our case, except for leukocytosis that along with symptoms such as fever and cough that lead us to the diagnosis of infections and pneumonia. In a similar study on a 32-year-old male who was diagnosed with IPT, lab data showed leukocytosis and on chest x-ray patient had a large pleural effusion in the lower two-third of the right lung which culture of the thoracentesis fluid grew *Salmonella enteritidis* [14]. Due to the severe adhesions during the operation, it’s possible that the IPT predisposed the patient to recurrent pneumonia of the left lung during the years. The fever and leukocytosis could be associated with the concomitant pneumonia of the patient.

Based on radiological findings, in the majority of the cases, cystic lesions often with focal calcification are reported [15] but in some cases, chest x-rays might be of non-diagnostic value, such as in the present study especially if calcified tissue such as bone and teeth are not present. Chest CT is considered as a standard technique of diagnosis as it reveals the exact location, extension, and the nature of the mass; however, it could also demonstrate non-specific findings [12]. Studies have shown a lobulated cystic structure with peripheral translucency is distinctive for the diagnosis of teratoma in CT scans. Furthermore, if air is observed in the mass, it could suggest the connection of the cyst to the bronchial tree [15].

The preoperative diagnosis in our case was in favor of empyema along with the possibility of hydatid cyst or lung abscess. Based on the nature of the tumor and symptoms, other possible differential diagnoses include ruptured hydatid cysts, fungal masses, lung abscess, pulmonary hamartoma, bronchogenic cyst, adenomatoid cystic malformation, intrapulmonary cystic lymphangioma, mediastinal teratoma, and pulmonary leiomyoma could also be considered [12, 13, 16–19]. In the discussed case, the preoperative CT scan implicated a misdiagnosis of a hydatid cyst in which the patient underwent anti-hydatidosis treatment. However, findings could be misleading if the diseases present with less common signs and symptoms.

Table 1 demonstrates a comparison of some typical features of the IPT case in our study with the possible differential diagnosis [10, 20–23].

**Table 1:** Clinical and radiological comparison of intrapulmonary teratoma, hydatid cyst of lung, and lung abscess

	Radiography	Signs and Symptoms	Involvement Features
**Intrapulmonary Teratoma**	• Typically cystic masses often with focal calcification and peripheral translucency • Air fluid level is suggestive of bronchial communication if present [9, 19]	• Chest pain • Hemoptysis • Cough • Trichoptysis (most specific) [19]	• Location: left upper lobe [9] • Unilateral [19]
**Hydatid cyst**	• Typically, a well-defined homogenous radio-opacity • Air fluid level in case of a complicated cyst [20]	• Usually asymptomatic for many years • Chest pain • Dyspnea • Dry cough • Hemoptysis [20]	• Location: lower lobes specially the right basal lobe • Bilateral in 20% of the cases [20]
**Acute Lung abscess** **(less than 6 week)**	• Usually circumscribed with not so well-defined surrounding to lung parenchyma • Air fluid level mostly present [21]	• Productive Cough • Fever • Night sweats [21]	• Location: posterior segments of the upper lobes and the superior segments of the lower lobes (if caused by aspiration) [21] • Usually unilateral [22]
**Chronic lung abscess**	• Usually irregular star-like shape with well-defined surrounding to lung parenchyma • Air fluid level mostly present [21]	• Productive Cough • Fever • Night sweats • Weight loss [21]	• Location: posterior segments of the upper lobes and the superior segments of the lower lobes (if caused by aspiration) [21] • Usually unilateral [22]

Rupture of the tumor, hemoptysis, airway compression, and malignant transformation are the complications of IPT if remains untreated [18, 24]. Surgery is considered as the optimal treatment and postoperative histopathological analysis provides the definitive diagnosis in which squamous epithelium with abundant keratin, connective tissues, components of fat tissue, calcifications such as teeth or bone, floating masses of hair and endometrial tissue could be observed [15, 25]. Similar characteristics have been presented in this case.

In conclusion, the preoperative diagnosis of IPT is not always possible and is usually misdiagnosed at first because of its rarity, non-specific and vague symptoms, normal laboratory results, and indistinguishable chest radiography findings. Initial diagnosis can be established based on a CT scan which can demonstrate calcification, cavitation, and peripheral translucency. Complete resection and surgery are considered as its gold standard curative treatment modality to avoid complications and malignant transformation***.*** Therefore, prompt diagnosis and suitable treatment should be immediately performed for these patients to avoid significant and life-threatening complications.

## Data Availability

Data of the patient can be requested from authors. Please write to the corresponding author if you are interested in such data.

## Abbreviations

IPT: Intrapulmonary teratoma
CT: Computed tomography
IV: Intravenous
